# Strategies for the Psychological Support of the Healthcare Workforce during the COVID-19 Pandemic: The ERNST Study

**DOI:** 10.3390/ijerph19095529

**Published:** 2022-05-02

**Authors:** Adriana López-Pineda, Irene Carrillo, Aurora Mula, Sofia Guerra-Paiva, Reinhard Strametz, Susanna Tella, Kris Vanhaecht, Massimiliano Panella, Bojana Knezevic, Marius-Ionut Ungureanu, Einav Srulovici, Sandra C. Buttigieg, Ivana Skoumalová, Paulo Sousa, Jose Mira, on behalf of the ERNST Consortium Collaborators

**Affiliations:** 1 The Foundation for the Promotion of Health and Biomedical Research of Valencia Region, 03550 Alicante, Spain; adriannalp@hotmail.com (A.L.-P.); amula@umh.es (A.M.); jose.mira@umh.es (J.M.); 2 Health Psychology Department, Miguel Hernandez University, 03202 Elche, Spain; 3 Public Health Research Centre, National School of Public Health, NOVA University of Lisbon, 1600-560 Lisbon, Portugal; sofiaguerrapaiva@gmail.com (S.G.-P.); paulo.sousa@ensp.unl.pt (P.S.); 4 Comprehensive Health Research Center (CHRC), 1600-560 Lisbon, Portugal; 5 Wiesbaden Business School, RheinMain University of Applied Science, 65183 Wiesbaden and German Coalition for Patient Safety, 10179 Berlin, Germany; reinhard.strametz@hs-rm.de; 6 Faculty of Social Services and Health Care, LAB University of Applied Sciences, 53850 Lappeenranta, Finland; susanna.tella@lab.fi; 7 Department of Quality, University of Leuven, 3000 Leuven, Belgium; kris.vanhaecht@kuleuven.be; 8 Department of Translational Medicine, University of Eastern Piedmont, 28100 Novara, Italy; massimiliano.panella@med.uniupo.it; 9 Department for Quality Assurance and Improvement in Healthcare, University Hospital Centre Zagreb, 10000 Zagreb, Croatia; bojana.knezevic@kbc-zagreb.hr; 10 Department of Public Health, Faculty of Political, Administrative and Communication Sciences, Babeș-Bolyai University, 400376 Cluj-Napoca, Romania; m.i.ungureanu@gmail.com; 11 Center for Health Workforce Research and Policy, Faculty of Political, Administrative and Communication Sciences, Babeș-Bolyai University, 400376 Cluj-Napoca, Romania; 12 The Cheryl Spencer Department of Nursing, University of Haifa, Haifa 3498838, Israel; esrulovici@univ.haifa.ac.il; 13 Department of Health Systems Management and Leadership, Faculty of Health Sciences, University of Malta, MSD 2080 Msida, Malta; sandra.buttigieg@um.edu.mt; 14 Department of Health Psychology and Research Methodology, Faculty of Medicine, Pavol Jozef Safarik University, 040 01 Košice, Slovakia; skoumalova.iva@gmail.com

**Keywords:** COVID-19, mental health, social support, health personnel, government programs

## Abstract

The COVID-19 pandemic led to the implementation of interventions to provide emotional and psychological support to healthcare workers in many countries. This ecological study aims to describe the strategies implemented in different countries to support healthcare professionals during the outbreak. Data were collected through an online survey about the measures to address the impact of the pandemic on the mental health of healthcare workers. Healthcare professionals, researchers, and academics were invited to respond to the survey. Fifty-six professionals from 35 countries contributed data to this study. Ten countries (28.6%) reported that they did not launch any national interventions. Both developed and developing countries launched similar initiatives. There was no relationship between the existence of any type of initiative in a country with the incidence, lethality, and mortality rates of the country due to COVID-19, and per capita income in 2020. The 24 h hotline for psychological support was the most frequent intervention. Tools for self-rescue by using apps or websites were extensively used, too. Other common interventions were the development of action protocols, availability of regular and updated information, implantation of distance learning systems, early detection of infection programs for professionals, economic reinforcements, hiring of staff reinforcement, and modification of leave and vacation dates.

## 1. Introduction

On 11 March 2020, the World Health Organization (WHO) declared COVID-19 a pandemic. More than a year later, 179,232,891 positive cases and 3,884,162 deaths have been reported globally, and 2,718,142,248 vaccine doses have been administered (23 June 2021) [1]. Over the past year, the pandemic has put health systems worldwide on the ropes, as they have had to take steps to cope with the extreme situation by making critical decisions on the fly.

Aside from the impact on the economy and population health, the pandemic has dealt a severe blow to the physical health and psychological well-being of healthcare professionals, who have worked tirelessly on the frontline in the fight against the virus. During the first wave, as of 8 May 2020, 152,888 infections and 1413 deaths had been reported in healthcare workers worldwide, with the former being more frequent among women and nurses and the latter among men and doctors [2]. Data published a few months later place the median worldwide mortality rate of healthcare professionals due to COVID-19 at 0.05 per 100,000 individuals [3]. In terms of the emotional impact, the most frequently observed psychological responses among healthcare professionals include distress (40–54%), anxiety (37–72%), depressive symptoms (38–53%), sleep disturbances (8–72%), and burnout (26–68%) [4]. The emotional distress experienced by healthcare professionals has led to the consideration of them as the second victims of the pandemic [5]. This disturbance constitutes a psychosocial hazard affecting employees’ health [6] and inevitably affects the quality of care provided to patients [7,8], making it necessary for healthcare systems to adopt measures to foster the resilience of health professionals and teams, and thus ensure institutional resilience [9].

The impact of the COVID-19 pandemic has reinforced the implementation of interventions to strengthen the resilience capacity of frontline health workers. The European Observatory on Health Systems and Policies reviewed the literature and identified 20 key strategies to enhance resilience during COVID-19 [10]. From this crisis, we have learned that maintaining an adequate healthcare workforce implies not only ensuring an adequate number of healthcare professionals, but also safeguarding the physical and mental abilities of each clinician to continue to care for a high volume of patients for a long time [11].

On 15 September 2020, the European Researchers’ Network Working on Second Victims (ERNST) Consortium was formally constituted [12]. This is a European Cooperation in Science and Technology (COST) Action (CA19113) currently involving 29 European countries and external collaborators from other countries (Azerbaijan, Japan, Latin America, and United States). The researchers adhering to this network are clinicians and academics from different disciplines who research in the field of patient safety and, specifically, the phenomenon of the second victim. During the pandemic, most of them have directly focused on interventions to support healthcare professionals caring for COVID-19 patients. The size and scope of this Consortium facilitate the exploration of the measures that have been adopted globally during the pandemic to promote the well-being and resilience of frontline healthcare workers. The primary aim of this study is to describe the different strategies that the member countries of the ERNST Consortium have launched at a national or local level (considering the political organization) to provide emotional and psychological support to healthcare professionals during the COVID-19 pandemic. The secondary objective is to determine the type of initiatives that were launched by a national entity according to the incidence, lethality, mortality rates of COVID-19, and the per capita income of the country.

## 2. Materials and Methods

This ecological study was conducted in the first semester of 2021 using data from 35 countries (86.5% of all member countries of the ERNST Consortium, *n* = 37). Data were collected through an online survey (Supplementary Data S1). Respondents were healthcare professionals, researchers, and academics who were participants of COST action CA19113 representing their countries [12], and with experience in health research or health management. In February 2021, they were invited via email, meeting, and personal contact to collaborate in this study completing the survey, and three reminders via email and social networks were sent to them for four months to improve the response rate. The participants were asked for consent before storing their answers. The subject matter of the study did not require approval by a research ethics committee.

The survey was developed by members of the research team (IC, RS, SG, JJM, RS, and ST), and consisted of 12 open- and closed-ended questions about the measures taken in the country to address the impact of this health crisis on the well-being and mental health of healthcare professionals. The interventions were either adopted at the national (i.e., launched or sponsored by the Ministry of Health or any national entity) or local (i.e., launched or sponsored by any specific regional entity) level. Interventions launched by healthcare institutions (hospitals, primary care centers, or others) for their workers were not considered. Two external investigators reviewed the questionnaire for legibility and understanding, and minor changes were required. The survey was performed using the Google Forms application and did not take more than 15 min to complete. All responses were downloaded directly into an Excel sheet.

Moreover, the following sociodemographic variables of respondents were collected: country; sex; age; professional profile (e.g., professor, researcher, physician, nurse, pharmacist, psychologist, midwife, other healthcare professionals, lawyer, statistician, sociologist, other legal, social, or technical profession); and type of work organization (e.g., higher education and associated organization, healthcare organization, government/intergovernmental organizations except for the Higher Education & Healthcare Organization, business enterprise, private non-profit without market revenues, NGO, standards organization). These data allowed checking the competence of respondents to provide data to the study.

The respondents had to answer all the survey questions to submit their responses, except for the open-ended questions. Several professionals from the same country were invited to respond to the survey to have as much information as possible. In the cases of missing data on the number of health workers infected with COVID-19 or deceased from COVID-19, the researchers searched for the information in reliable online sources.

The information/data about the impact of the pandemic in each country (incidence, lethality, and mortality) was retrieved from the Coronavirus Resource Center of Johns Hopkins [1] on 5 March 2021 (data updated as of 5 March 2021). In addition, the per capita income data for 2020 were extracted from the Datosmacro website [13].

### Data Analysis

The frequencies and percentages were calculated for responses to closed-ended questions. Answers to open-ended questions were analyzed following the steps of the framework analysis [14]. Firstly, four members of the research team (ALP, IC, AM, and JJM) read the responses and generated initial categories once the key ideas from the data were identified. This reading yielded a classification of the initiatives available across countries and allowed indexing the interventions to prevent distress in healthcare workers in a Microsoft Office 2021 Excel Spreadsheet (Microsoft, Redmond, WA, USA). Vague or generic responses that were not referring to a specific intervention were not codified. Responses to open-ended questions that we considered did not answer the question were removed. Finally, research questions were used as an analytic guide for understanding the data. The research team discussed these results before finally developing a conceptual explanation of the data.

For the secondary objective, the initiatives on which the respondents were asked the level of implementation in their country through closed questions (items 6, 7, 8, and 9 of the questionnaire) were reclassified into 3 blocks: initiatives to improve working conditions, initiatives for psycho-emotional reinforcement and support, and initiatives to strengthen staff, teams, and the organization (Appendix A). In addition, each country was classified according to its incidence, lethality (or case fatality), and mortality rates, and to their per capita income in 2020. To identify three levels, the distributions and quartiles of these variables were obtained. The following ranges were considered: low (≤5500 cases per 100,000 inhabitants), medium (>5500 and <8000 cases per 100,000 inhabitants), and high (≥8000 cases per 100,000 inhabitants) incidence; low (≤2 deaths per 100 cases), medium (>2 and <3 deaths per 100 cases), and high (≥3 deaths per 100 cases) lethality; low (≤100 deaths per 100,000 inhabitants), medium (>100 and <179 deaths per 100,000 inhabitants) and high (≥179 deaths per 100,000 inhabitants) mortality; and low (≤EUR 8000), medium (>EUR 8000 and <EUR 20,000), and high (≥EUR20,000) per capita income. The Fisher test was used to analyze the relationship between the existence of any type of initiative in a country with the incidence, lethality, and mortality rates of the country due to COVID-19, and per capita income in 2020. In addition, a hierarchical cluster analysis based on squared Euclidean distances was performed with the following study variables: incidence; lethality and mortality rates; per capita income; any initiative at national level (yes/no); initiatives to improve working conditions (yes/no); initiatives for psycho-emotional reinforcement and support (yes/no); and initiatives to strengthen staff, teams, and the organization (yes/no). The SPSS software version 22.0 was used to analyze the data.

## 3. Results

Fifty-six professionals from 35 countries contributed data to this study. The response rate was 41.2%, since 136 professionals were invited to respond. Table 1 shows the characteristics of respondents and they are all professionals linked to the health area, affiliated with an institution that addresses patient care, health research, health management, or professional welfare. Table 2 discloses the included countries and the information on the number of healthcare workers who were infected with COVID-19 and data on the number of professionals who died from COVID-19. On the date of data collection, these data were only available in some countries. For some countries, there was no published official data.

Table 3 shows the countries that launched any initiative to provide emotional and psychological support to healthcare professionals and reduce their stress levels at the national level (responses to open-ended question number 4 of the survey). These initiatives especially included psychological support for HCWs by professionals at any time and social recognition, defined as social events among the population or any form to make known that they appreciate the work conducted by healthcare workers during the pandemic, for example, and applause every day, thank you videos on audiovisual media or on social networks. Seven countries (20%) that participated in this study reported that no initiative was launched in their countries at the national level. The incidence, lethality, mortality rates, and per capita incomes of each country are shown in Table 3. The red, yellow, and green colors represent high, medium, and low levels, respectively. Although no statistically significant association was found, 9 out of 10 countries with a high level of incidence had launched some nationwide support strategy. There was also no association between having nationwide support strategies and the level of lethality or mortality in the country due to COVID-19 (*p* > 0.05). Table S1 shows the contingency tables. Likewise, the per capita income of the country was not associated with the launching of support initiatives by a national entity of the country (*p* > 0.05). The dendrogram in Figure S1 shows the clusters identified in the average linkage clustering and any relevant cluster was identified.

Additionally, the association between the fact of implementing any initiative of the proposed strategy blocks and the impact of the pandemic (incidence, lethality, and mortality rate) and the per capita income of the country were assessed. However, at least one initiative of each strategy block was implemented at the national and/or local level in all countries.

**Figure 1:**
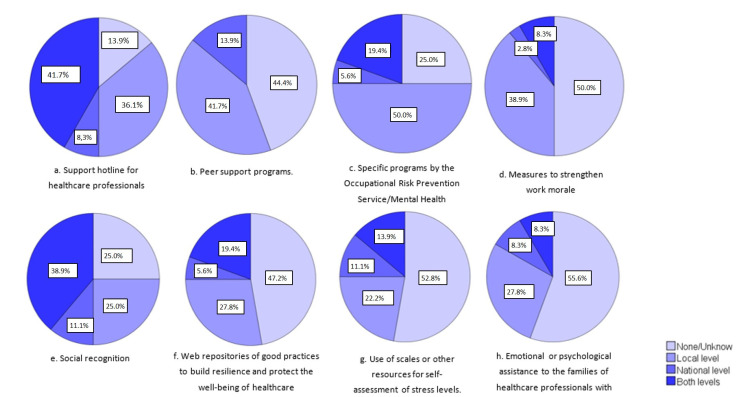
Initiatives to provide emotional or psychological support to health professionals from 35 countries (Argentina, Azerbaijan, Belgium, Bosnia and Herzegovina, Brazil, Bulgaria, Chile, Colombia, Croatia, Czech Republic, Denmark, Ecuador, Estonia, Finland, France, Germany, Ireland, Israel, Italy, Japan, Lithuania, Malta, Mexico, Netherlands, Peru, Poland, Portugal, Romania, Serbia, Slovakia, Spain, Sweden, Switzerland, Turkey, and the United States).

**Figure 2:**
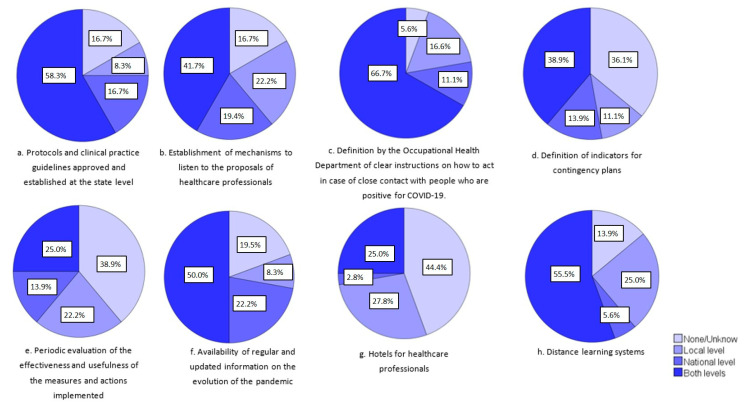
Organizational initiatives to respond to the challenges of the COVID-19 pandemic effectively from 35 countries (Argentina, Azerbaijan, Belgium, Bosnia and Herzegovina, Brazil, Bulgaria, Chile, Colombia, Croatia, Czech Republic, Denmark, Ecuador, Estonia, Finland, France, Germany, Ireland, Israel, Italy, Japan, Lithuania, Malta, Mexico, Netherlands, Peru, Poland, Portugal, Romania, Serbia, Slovakia, Spain, Sweden, Switzerland, Turkey, and the United States).

**Figure 3:**
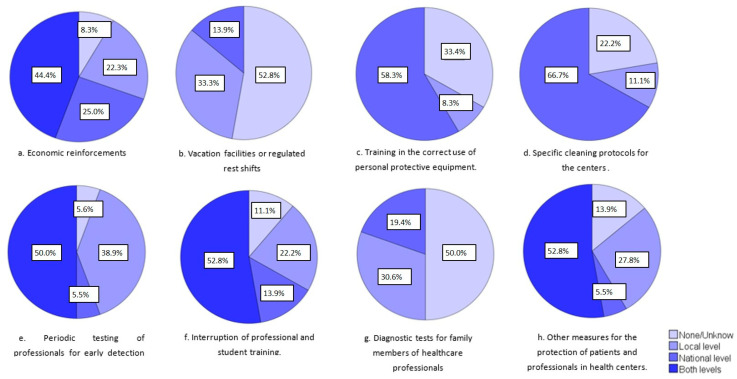
Initiatives to improve working and safety conditions and reward the efforts of healthcare professionals from 35 countries (Argentina, Azerbaijan, Belgium, Bosnia and Herzegovina, Brazil, Bulgaria, Chile, Colombia, Croatia, Czech Republic, Denmark, Ecuador, Estonia, Finland, France, Germany, Ireland, Israel, Italy, Japan, Lithuania, Malta, Mexico, Netherlands, Peru, Poland, Portugal, Romania, Serbia, Slovakia, Spain, Sweden, Switzerland, Turkey, and the United States).

**Figure 4:**
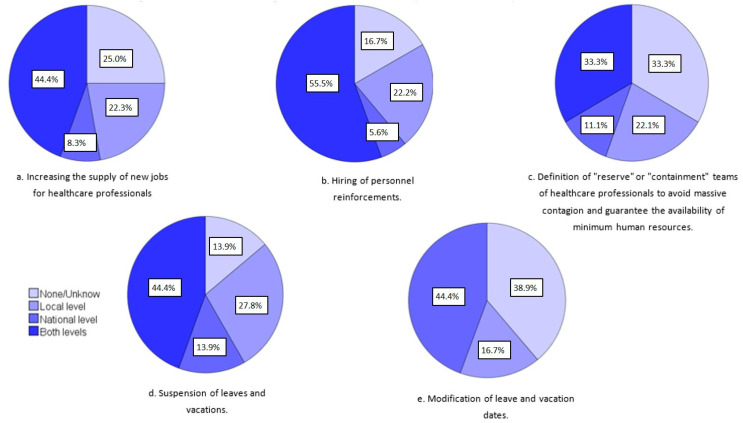
Initiatives to maintain or strengthen the human resources and workforce of health institutions from 35 countries (Argentina, Azerbaijan, Belgium, Bosnia and Herzegovina, Brazil, Bulgaria, Chile, Colombia, Croatia, Czech Republic, Denmark, Ecuador, Estonia, Finland, France, Germany, Ireland, Israel, Italy, Japan, Lithuania, Malta, Mexico, Netherlands, Peru, Poland, Portugal, Romania, Serbia, Slovakia, Spain, Sweden, Switzerland, Turkey, and the United States).

Figure 1, Figure 2, Figure 3 and Figure 4 show the results of the answers to closed-ended questions number 6, 7, 8, and 9 of the survey (Supplementary Data S1). Concerning the initiatives to provide emotional or psychological support to health professionals, Figure 1 shows the proportion of countries that launched some type of initiative for this proposal at the national or local levels, or both. The most implemented initiatives at the national and local levels were the support hotline for healthcare professionals and social recognition. Specific programs developed by the occupational risk-prevention service, mental health service, or employee assistance unit were also launched by most countries at the local level.

In relation to the organizational initiatives to respond to the challenges of the COVID-19 pandemic effectively, Figure 2 shows the proportion of countries that launched some type of initiative for this proposal at the national level, local level, or both. The definition and establishment by the occupational health department of clear instructions on how to act in case of close contact with people who are positive for COVID-19, hospitalization, and discharge of COVID-19 patients, and the distance learning systems were the most implemented strategies. According to the information reported by the respondents, 36.1% of the countries did not define indicators for contingency plans and 38.9% did not periodically evaluate the effectiveness and usefulness of the measures and actions implemented, or these actions were not known.

In regard to the initiatives to improve the working and safety conditions and reward the efforts of healthcare professionals, Figure 3 shows the proportion of countries that launched some type of initiative for this proposal at the national level, local level, or both. Economic reinforcements, the periodic testing of professionals for the early detection of infection, and the interruption of professional and student training were the most implemented actions by the countries at both levels.

In consideration of the initiatives to maintain or strengthen the human resources and workforce of health institutions, Figure 4 shows the proportion of countries that launched some type of initiative for this proposal at the national level, local level, or both. The modification of leave and vacation dates and the hiring of personnel reinforcements were the actions most implemented by the countries. Twenty-five (44.6%) respondents thought that many of these initiatives declined over time.

A total of 6 of the 35 (17.1%) countries (Israel, Lithuania, Mexico, Peru, Serbia, and Turkey) classified the infections among healthcare workers during the rest periods of their shifts as a social outbreak, i.e., in these countries, the health professional was not considered to have been infected in the course of his/her professional work. In contrast, Argentina, Azerbaijan, Brazil, Bulgaria, Chile, Colombia, Croatia, Czech Republic, Ecuador, Germany, Italy, Malta, Netherlands, Peru, Poland, Romania, Serbia, Slovakia, Spain, Turkey, and the United States (60.0%) classified these infections as a professional outbreak, i.e., they considered that these infections were associated with the exercise of care activities. The rest of the collaborators answered not knowing this information about their country, because this issue might not have been raised in the national debate or equivalent.

In consideration of the specific measures used to enhance the emotional recovery of healthcare professionals in periods of remission of the epidemic (question number 5 of the survey), Argentina, Belgium, Lithuania, Netherlands, Portugal, Slovakia, Spain, and the United States answered that “Yes”, they have taken measures. Fifteen countries (42.9%) did not take this type of measure and this information was unknown for the rest of the countries.

## 4. Discussion

The responses to support healthcare professionals during the first period of the pandemic in each country were different, although there were certain initiatives, such as psychological support for HCWs, which were common and widely used in many of the countries. They included interventions to enhance the individual capacity of the professionals to face the overload due to the rocketing incidence of COVID-19 from the first days of the pandemic in each country. In addition, the respondents also reported the interventions that were focused on the healthcare system as a whole to assure an appropriate response to patient needs (either COVID-19 or non-COVID-19 patients). The results of the present study suggest that all these interventions have been implemented, regardless of COVID-19 incidence, mortality, or lethality. In addition, both developed and developing countries have launched similar initiatives.

Healthcare systems have had to address both the loss of professionals due to extreme fatigue and distress and the disintegration of work teams due to overload and the relocation of professionals to other healthcare units [21].

The so-called primary psychological aid was offered to healthcare workers in practically all the countries analyzed in this study. Other interventions have been aimed at promoting the activation of natural protective factors of acute stress (information feedback, feeling supported by society, rest times, self-assessment of stress, and hotels for professionals), e.g., reinforcing resilience. These measures, for the most part, have been oriented toward a secondary prevention of the effects of the pandemic on professionals. The pandemic has had a rapid and intense impact on healthcare professionals without the opportunity of healthcare professionals preparing for this. Secondary prevention was promoted from the first weeks of the pandemic to reduce the impact of the pandemic on the entire workforce.

The 24 h hotline service for psychological first aid has been the most frequent intervention to address the impact of the first wave of the COVID-19 pandemic on healthcare workers in the majority of countries. Other frequent nationwide initiatives to support them have included constant information about the pandemic evolution, social recognition, peer support programs, and web repositories with resources to enhance individual responses to distress and resources of good practices. In some countries, national or local interventions included residential facilities to avoid returning home after the work shift and to support healthcare professionals’ relatives. All these interventions have in common that their implementation has a low level of complexity and do not require interrupting the activity in the health centers, and, therefore, can be used by many professionals. Previous studies found that the interventions put in place during this period to address the impact of the pandemic on healthcare workers have been similar to those described here [22,23,24]. Psychological counseling and measures to prevent burnout have been described by professionals as the most common interventions to support them [25]. However, the professionals themselves decided to use the tools made available to them or did not consider it necessary for them. The fact of deciding by themselves if they needed such a tool constitutes the main barrier, since we know that many of them did not recognize that they were affected by the overload until after the pressure of healthcare decreased [26].

The COVID-19 pandemic has generated an unprecedented use of self-rescue tools. For the first time, there has been an extensive use of electronic resources (e.g., apps and websites) to deal with the distress experienced by healthcare workers. The positive value of timely and clear messages about the infection figures, together with the ultimate goal of managing the pandemic, has helped healthcare teams to cope with this crisis and increase their responsiveness to the work teams [6]. Other interventions described in the literature and the present study have sought to improve top–bottom communication channels to offer pandemic updates, and bottom-up communication to learn what professionals need, e.g., rest and time off for recovery workers, assure sufficient supplies of adequate protective equipment, or alternative accommodation to reduce the fear of infecting families when they come back home [6,22,23]. The role of the Occupational Health Department in the elaboration of protocols, the open spaces to listen to what professionals had to say, and the training to cope with distress have been highlighted.

Unlike the support initiatives, which were adopted to varying degrees in all countries, the improvement of work conditions was less consistently reported. The results of this study show that the training in the proper use of personal protective equipment has been intensified and the cleaning procedures have been reinforced. Additionally, the study countries strengthened the capacity for the early detection of infections among healthcare professionals. In almost half of the countries, economic incentives have been used to support frontline healthcare professionals. Very few countries offered rest periods to their staff to recover from the overload. On the contrary, most countries suspended leave and vacations of their staff. This might come as a surprise, since this strategy contradicts the objective of maintaining healthy workers and workplaces, which is essential during pandemics [27]. Most countries also proceeded to urgently recruit new staff to reinforce the work teams.

In regard to the planned internships for students and trainees in healthcare centers being suspended, this interruption meant that their rotation was suspended and the knowledge and skills to be acquired during this period were not learnt. The impact of this suspension has not been evaluated, and no specific interventions have been addressed for them. The administrative staff of the healthcare institutions assumed a critical role during the pandemic. Although support initiatives targeting frontline HCWs have proliferated, the emotional and psychological needs of administrative staff have hardly been addressed [28]. Almost half of the study respondents thought that many of the support initiatives declined over time; the reason might be the priority vaccination of professionals.

The measures that have been described are directly related to how to deal with the impact of the pandemic on HCWs. It remains to be seen which interventions and actions are put in place to recover the health systems from the effect of COVID-19 and to support the workforce in facing the new health challenges, such as the recovery of the suspended activity during this period [29,30].

### 4.1. Strengths and Study Limitations

This study offers an ample vision of comparative approaches and interventions across multiple countries that are not usually published in this manner. The study findings reveal that the solutions were similar around the world, and they were based on already available interventions and similar to those used in other previous health crises. Moreover, it highlights how most national authorities have recognized the importance of considering the well-being of healthcare professionals for the optimal care of patients. The study findings are limited to the point in time and phase of the pandemic when the data were collected. It does not measured the effectiveness of the available measures and resources focused on addressing the impact of the pandemic on healthcare professionals. These data are based on participants’ reports. Although many informants provided sources of information (such as reports and websites), other relevant information may not have been reported. Interventions to support professionals that have been carried out at the level of health centers for their staff have not been considered in this study.

### 4.2. Implications for Policy, Practice, and Research

Those support interventions that could be activated more rapidly and extended to a large number of professionals were implemented in all countries, regardless of their income level. Since almost no country has monitored the usefulness of the interventions, a future assessment of the usefulness of these interventions seems advisable. The measure of their real impact would allow us to activate those that have the best results and acceptability among workers in the future. However, such evaluations are not possible, since many of the interventions have diminished, even as the pandemic continues.

Some of the interventions, such as communication channels, may continue to be useful for improving the conditions in which healthcare professionals work. Similarly, the resources aimed at reducing professional stress, which is otherwise common in these professions, could be maintained to contribute to increasing the resilience of professionals, which would probably have a positive impact on the quality of healthcare received by patients.

Future research could draw lessons learned from great enormous effort to establish recommendations and guidelines for action in future outbreaks, since there is a clear need to strengthen the resilience of professionals in the face of crisis situations of this magnitude to reduce depression, stress, burnout, relationship problems, suicidal ideation, alcohol abuse, performance problems, or intentions to quit [31,32,33].

## 5. Conclusions

Among the strategies to support healthcare professionals during the outbreak launched by countries of the ERNST Consortium, the 24 h hotline for psychological support was the most frequent intervention. Tools for self-rescue by using apps or websites were extensively used, too. Other common interventions were the development of action protocols, availability of regular and updated information, implantation of distance-learning systems, early detection of infection programs for professionals, economic reinforcements, hiring of staff reinforcement, and modification of leave and vacation dates. No association between having nationwide support strategies and the level of lethality or mortality in the country due to COVID-19 was found. Similarly, no association was noted between the per capita income of the country and the launching of support initiatives.

## Figures and Tables

**Table 1:** Characteristics of the respondents (*n* = 56).

Sociodemographic Variables	
Sex (*n*, %)	
Female	36 (64.3%)
Male	20 (35.7%)
Age (mean, [min–max])	47.0 [24–68]
Professional profile (*n*, %)	
Nurse ^1^	5 (8.9%)
Physician ^1^	18 (32.1%)
Psychologist ^1^	2 (3.6%)
Other healthcare professionals	1 (1.8%)
Researchers on public health or related disciplines	13 (23.2%)
Professor in the health area	15 (26.8%)
Other legal, social, or technical profession	2 (3.6%)
Type of work organization (*n*, %)	
Healthcare organization	21 (37.5%)
Higher education and associated organizations	27 (48.2%)
Government/intergovernmental organizations except higher education and healthcare organization	3 (5.4%)
Private non-profit without market revenues, NGO	2 (3.6%)

^1^ Working in the healthcare system.

**Table 2:** The number of healthcare workers (HCWs) who were infected with COVID-19 and the number of HCWs who died from COVID-19.

Country	Number of HCWs Infected with COVID-19	Number of HCWs who Died from COVID-19	Date of Data Obtainment	Data Source
Argentina ^1^	79,806	472	March 2021	Ministerio de Sanidad de Argentina
Azerbaijan ^2^	No data	45	July–August 2020	Erdem and Lucey [3]
Belgium ^3^	No data	No data		
Bosnia and Herzegovina ^3^	2019	8	April 2021	Provided by the collaborator
Brazil ^1^	48,234 nurses	556 nurses	February 2021	Provided by the collaborator
Bulgaria ^3^	12670	No data	April 2021	Provided by the collaborator
Chile ^1^	52,241	102	January 2021	Epidemiological report from the Ministry of Health [15]
Colombia ^1^	3655	31	July–August 2020	Erdem and Lucey [3]
Croatia ^3^	2551 physicians	2 physicians	February 2021	Croatian Medical Chamber [16]
Czech Republic ^3^	1119	2	July–August 2020	Erdem and Lucey [3]
Denmark ^3^	2380	15	March 2021	Statens Serum Institut [17]
Ecuador ^1^	No data	No data		
Estonia ^3^	No data	No data		
Finland ^3^	2000	No data	January 2021	Provided by the collaborator
France ^3^	81,032	16	May 2021	French public health [18]
Germany ^3^	104,387	148	March 2021	Provided by the collaborator
Ireland ^3^	16,381	9	April 2021	Provided by the collaborator
Israel ^4^	No data	No data		
Italy ^3^	138,129	333	July 2021	Ministry of Health from Italy
Japan ^1^	No data	No data		
Lithuania ^3^	No data	No data		
Malta ^3^	No data	No data		
Mexico ^1^	No data	No data		
Netherlands ^3^	No data	No data		
Peru ^1^	13,073 physicians ^a^ + 7780 nurses ^b^	400 physicians ^a^ + 90 nurses ^b^	^a^ March 2021 ^b^ January 2021	Provided by the collaborator
Poland ^3^	91,949	187	February 2021	Provided by the collaborator
Portugal ^3^	3681	1	July–August 2020	Erdem and Lucey [3]
Romania ^3^	20,504	94		Provided by the collaborator
Serbia ^3^	No data	No data		
Slovakia ^3^	No data	40	September 2021	Institute of Health Analysis (Ministry of Health)
Spain ^3^	128,590 ^c^	112 ^d^	^c^ February 2021 ^d^ March 2021	^c^ Ministerio de Sanidad de España [19] ^d^ Consejo General de Colegios Oficiales de Médicos [20]
Sweden ^3^	No data	No data		
Switzerland ^3^	No data	No data		
Turkey ^3^	>120,000	383	January 2021	Provided by the collaborator
United States ^1^	114,529	574	July–August 2020	Erdem and Lucey [3]

^1^ COST International Partner Countries; ^2^ COST Near Neighbor Countries; ^3^ COST member; and ^4^ Cooperating member. The date of data obtainment is different in case of physicians or nurses. “a” means the date of data obtainment in case of physicians and “b” means the date of data obtainment for nurses. “c” means the date of data obtainment for Number of HCWs Infected with COVID-19. “d” means the data on number of HCWs who died in Spain (*n* = 112) was updated on March 2021 and the data source was Consejo General de Colegios Oficiales de Médicos [20].

**Table 3:** Initiatives to provide emotional and psychological support to healthcare professionals at the national level and reduce their stress levels, and the incidence, lethality (or case fatality), mortality rates due to COVID-19 (data updated as of 5 March 2021) and the per capita incomes (2020) of each country.

Country	Initiatives at National Level	Incidence (per 100,000 Inhabitants)	Lethality (%)	Mortality (per 100,000 Inhabitants)	Per Capita Income (EUR )
Argentina	Emotional support with online inquiries and helpline by a net of psychologists (Argentine Society of Intensive Care, only to their partners).	6589.6	2.1	140.9	EUR 7463
Azerbaijan	Support hotline for healthcare workers, peer support programs, measures to strengthen work morale, social recognition, and self-assessment of stress level.	3143.6	1.4	44.6	EUR 4263
Belgium	Website, webinars, and research (example: www.dezorgsamen.be, accessed on 15 September 2021)	8558.6	2.4	208.8	EUR 39,110
Bosnia and Herzegovina	Support hotline for healthcare workers.	6081.3	4.3	262.0	EUR 5266
Brazil	No support interventions, but there is social recognition.	6895.0	2.8	190.5	EUR 6013
Bulgaria	No.	5875.2	4.1	239.1	EUR 8750
Chile	No support interventions, but there is social recognition.	6302.8	2.2	138.2	EUR 11,582
Colombia	Recommendations.	5644.4	2.6	145.3	EUR 4718
Croatia	For the prevention of “burn-out syndrome”: 24 h hotline for psychological help for healthcare workers (Croatian Institute for Public Health).	8220.2	2.2	176.8	EUR 12,170
Czech Republic	Healthcare insurance company partially covers psychotherapy.	15,241.8	1.8	273.8	EUR 19,970
Denmark	No.	3693.4	1.1	41.0	EUR 53,470
Ecuador	No.	2165.1	4.8	104.8	EUR 5592
Estonia	No support interventions, but there is social recognition and the use of scales and other resources for the self-assessment of stress levels.	5438.5	0.9	48.0	EUR 20,440
Finland	Support hotline for healthcare workers.	1574.2	1.0	16.5	EUR 42,940
France	National hotline for psychological support (regional medico-psychological emergency unit).	8732.6	1.8	160.5	EUR 34,040
Germany	No.	4094.4	2.4	99.3	EUR 40,120
Ireland	Psychological support via professional organizations.	5013.9	2.0	98.5	EUR 73,590
Israel	A 24 h hotline for support by psychologists and social workers (Ministry of Health).	8799.7	0.7	64.5	EUR 38,942
Italy	INAIL (National Institute of Workers’ Compensation—Insurance) with the psychologist council activated on a 24 h support telephone line. Taskforce for developing guidelines for the local implementation of psychological support from the HCW webpage.	6700.2	3.0	201.2	EUR 27,780
Japan	Telephone-based consultation initiative for nurses on the frontlines (Japan Nurses’ Association).	339.1	1.9	6.4	EUR 35,059
Lithuania	Psychologist consultation for healthcare workers (Ministry of Health).	9306.7	1.6	147.1	EUR 17,510
Malta	Psychology Department Mater Dei Hospital website in Facebook.	4535.6	1.4	62.3	EUR 25,310
Mexico	(1) Government initiatives: Webpage with some support resources, including mindfulness audio, some videos, a list of emotional support initiatives, and a mental-health-risks questionnaire. (2) University initiatives: Community-exclusive call centers and a telepsychiatry clinic. (3) Civil societies: Telepsychiatry for health professionals, and a list of emotional support networks	1803.2	9.2	166.8	EUR 7379
Netherlands	Websites for information consultation. For example, the Trimbos Institute. Impact Kenniscentrum.	8987.6	1.1	101.6	EUR 45,870
Peru	Phone number by the government for support. Webinars for health workers by psychologists and psychiatrists. Friend call for psychological support. Rapid-response teams. Psychosocial support.	5428.7	3.4	186.2	EUR 5488
Poland	No support interventions, but there is social recognition.	7423.2	2.4	180.2	EUR 13,600
Portugal	Psychological counseling from the national health service to support the psychological concerns and challenges of patients and health professionals (who are providing health care).	8236.3	2.0	167.0	EUR 19,660
Romania	No.	5533.0	2.7	148.4	EUR 11,290
Serbia	No support interventions, but there is social recognition.	7969.7	0.9	74.0	EUR 6710
Slovakia	Psycho-social support teams during the 1st pandemic wave (Ministry of Health). Online application to monitor the stages of depression and anxiety via a questionnaire and to provide recommendations.	7017.9	3.1	216.2	EUR 16,770
Spain	Telephone support from the Ministry of Health.	7538.9	2.2	167.3	EUR 23,690
Sweden	No.	9582.6	1.4	138.3	EUR 45,850
Switzerland	No.	7618.1	1.6	122.2	EUR 75,890
Turkey	RUHSAD (mental health support system) app to offer psychosocial counseling (over the telephone or online systems) and mental health support services.	5732.9	0.8	48.0	EUR 7520
United States	Web repositories of good practices to build resilience and protect the well-being of healthcare professionals, and the use of scales or other resources for the self-assessment of stress levels.	9739.4	1.8	173.3	EUR 55,806

Categories: high (red), medium, (yellow), and low (green) levels. Sources of data: Coronavirus Resource Center of Johns Hopkins [1] and the Datosmacro website [13], accessed on 5 March 2021.

## Data Availability

The data that support the findings of this study are available from the corresponding author, I.C., upon reasonable request.

## Supplementary Materials

The following supporting information can be downloaded at: https://www.mdpi.com/article/10.3390/ijerph19095529/s1, Supplementary Data S1: Online survey to obtain the study information; Table S1: Contingency tables of the association between the existence of initiatives at national level and the impact of COVID-19; Figure S1: Dendrogram of hierarchical cluster analysis based on squared Euclidean distances using average linkage between groups.

## Appendix A

Re-classification of the initiatives into three blocks:

1. Initiatives to improve working conditions:
  - Economic reinforcements, such as payments and salary increases.
  - Vacation facilities or regulated rest shifts.
  - Training in the correct use of personal protective equipment.
  - Specific cleaning protocols for the centers.
  - Periodic testing of professionals for the early detection of infection.
  - Interruption of professional and student training.
  - Diagnostic tests for the family members of healthcare professionals.
  - Other measures for the protection of patients and professionals in health centers (for example, triage and change of mask for everyone who enters the center).
  - Hotels for healthcare professionals (or other accommodation options to avoid the risk of contagion to family members and loved ones).
  - Modification of leave and vacation dates.
2. Initiatives for psycho-emotional reinforcement and support:
  - Support hotline for healthcare professionals.
  - Peer support programs.
  - Specific programs developed by the Occupational Risk Prevention Service/Mental Health Service/Employee Assistance.
  - Measures to strengthen work morale.
  - Social recognition.
  - Web repositories of good practices to build resilience and protect the well-being of healthcare professionals.
  - Use of scales or other resources for the self-assessment of stress levels.
  - Emotional or psychological assistance to the families of healthcare professionals with COVID-19.
3. Initiatives to strengthen staff, teams, and the organization:
  - Increasing the supply of new jobs for healthcare professionals.
  - Hiring of personnel reinforcements.
  - Definition of “reserve” or “containment” teams of healthcare professionals to avoid massive contagion and guarantee the availability of minimum human resources.
  - Suspension of leaves and vacations.
  - Modification of leave and vacation dates.
  - Protocols and clinical practice guidelines approved and established at the state level by the Ministry of Health and other national entities (shared action criteria among regions and health institutions in the country).
  - Establishment of mechanisms to listen to the proposals of healthcare professionals (e.g., working groups with professional associations, trade unions, and scientific societies).
  - Definition and establishment by the Occupational Health Department of clear instructions on how to act in case of close contact with people who are positive for COVID-19, hospitalization, and discharge of COVID-19 patients.
  - Definition of the indicators for the contingency plans.
  - Periodic evaluation of the effectiveness and usefulness of the measures and actions implemented.
  - Availability of regular and updated information on the evolution of the pandemic at national, regional, and local levels to healthcare professionals (including data on infected and deceased healthcare professionals).
  - Distance learning systems (e.g., healthcare professionals in home isolation who advise and train new personnel, training in the use of personal protective equipment, and new protocols and practices)
