# Supplementary material for: Strategies for the Psychological Support of the Healthcare Workforce during the COVID-19 Pandemic: The ERNST Study
Source: Int J Environ Res Public Health. 2022 May 2;19(9):5529. doi: 10.3390/ijerph19095529 (PMC9105489; doi:10.3390/ijerph19095529)
Supplement: Supplementary file 1 [file ijerph-19-05529-s001.zip › ijerph-1660301-supplementary.pdf]

Supplemental Data 1: Online survey to obtain the study information.

1. The number of healthcare professionals infected with COVID-19 in your country (Date and information source).
2. The number of healthcare professionals who have died from COVID-19 in your country (Date and information source).
3. What classification has been given to infections among health professionals during rest periods in their shifts?
4. Any initiative launched or sponsored by the Ministries of Health or other national entities, to provide emotional and psychological support to healthcare professionals and reduce their stress levels (Type and information source).
5. In periods of remission of the epidemic in your country (between waves), have specific measures been put in place to enhance the emotional recovery of healthcare professionals?
6. The level of implementation of the following <u>initiatives to provide emotional or psychological support to health professionals</u> (national level/local level/both levels/none, I don't know):
    a. Support hotline for healthcare professionals
    b. Peer support programs.
    c. Specific programs developed by the Occupational Risk Prevention Service/Mental Health Service/Employee Assistance
    d. Measures to strengthen work morale
    e. Social recognition
    f. Web repositories of good practices to build resilience and protect the well-being of healthcare professionals
    g. Use of scales or other resources for self-assessment of stress levels.
    h. Emotional or psychological assistance to the families of healthcare professionals with COVID-19.
7. The level of implementation of the following <u>organizational initiatives to effectively respond to the challenges posed by the SARS-CoV-2 pandemic</u> (national level/local level/both levels/none, I don't know):

   a. Protocols and clinical practice guidelines approved and established at the state level by the Ministry of Health and other national entities (shared action criteria among regions and health institutions in the country).
   b. Establishment of mechanisms to listen to the proposals of healthcare professionals (e.g., working groups with professional associations, trade unions and scientific societies).
   c. Definition and establishment by the Occupational Health Department of clear instructions on how to act in case of close contact with people who are positive for COVID-19, hospitalization, and discharge of COVID-19 patients, etc.
   d. Definition of indicators for contingency plans
   e. Periodic evaluation of the effectiveness and usefulness of the measures and actions implemented
   f. Availability of regular and updated information on the evolution of the pandemic at national, regional, and local levels to healthcare professionals (including data on infected and deceased healthcare professionals)
   g. Hotels for healthcare professionals (or other accommodation options to avoid the risk of contagion to family members and loved ones).]
   h. Distance learning systems (e.g., healthcare professionals in home isolation who advise and train new personnel, training in the use of personal protective equipment, new protocols, and practices, etc.)
8. The level of implementation of the following <u>initiatives to improve working and safety conditions and reward the efforts of healthcare professionals</u>:
   a. Economic reinforcements. Extra payments, salary increases, etc.
   b. Vacation facilities or regulated rest shifts.
   c. Training in the correct use of personal protective equipment.
   d. Specific cleaning protocols for the centers.
   e. Periodic testing of professionals for early detection of infection.
   f. Interruption of professional and student training.
   g. Diagnostic tests for family members of healthcare professionals.

h. Other measures for the protection of patients and professionals in health centers (for example, triage and change of mask for everyone who enters the center).

9. The level of implementation of the following initiatives to maintain or strengthen the human resources and workforce of health institutions:
   a. Increasing the supply of new jobs for healthcare professionals
   b. Hiring of personnel reinforcements.
   c. Definition of "reserve" or "containment" teams of healthcare professionals to avoid massive contagion and guarantee the availability of minimum human resources.
   d. Suspension of leaves and vacations.
   e. Modification of leave and vacation dates.
10. To describe the initiatives checked in the previous questions or provide any additional information that may be of interest.
11. Has the implementation of the initiatives you have marked above been sustained over time and waves or has it been declining?
12. What other initiatives, so far not mentioned, do you consider appropriate to implement to reduce the impact of the pandemic on the well-being and mental health of healthcare professionals

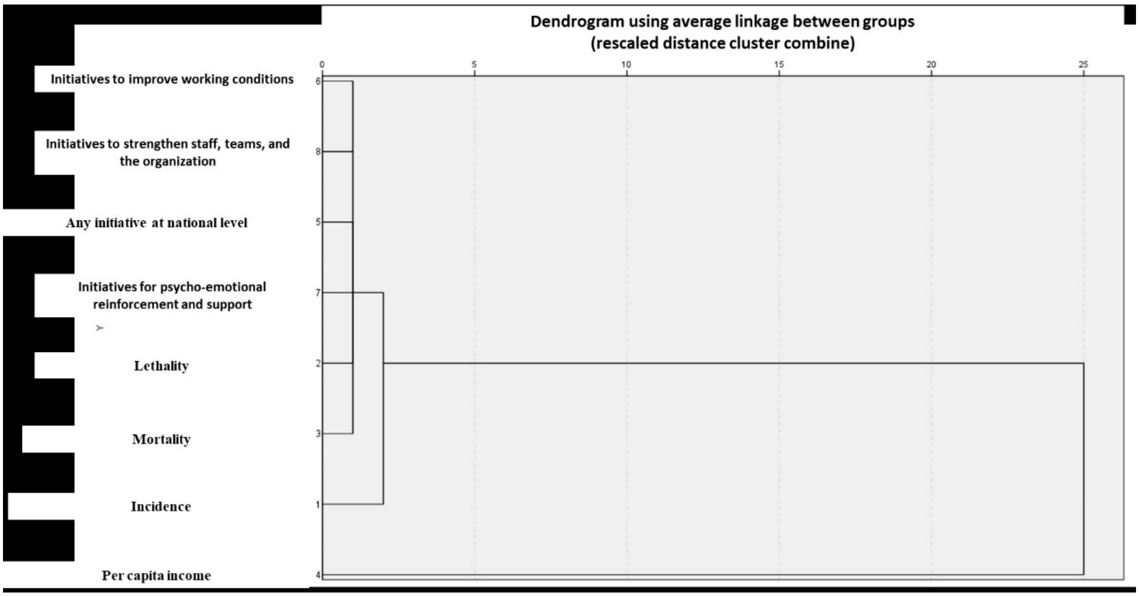

Figure S1: Dendrogram of hierarchical cluster analysis based on squared Euclidean distances using average linkage between groups.

Table S1. Contingency tables of the association between the existence of initiatives at national level and the impact of COVID-19.

Initiatives	Low incidence (n,%)	Medium incidence (n,%)	High incidence (n,%)	Total (n,%)
No	3 (27.3%)	6 (42.9%)	1 (10%	10 (28.6%)
Yes	8 (72.7%)	8 (57.1%)	9 (90%)	25 (71.4%)
Total	11 (100%)	14 (100%)	10 (100%)	35 (100%)

Fisher's p = 0.240

Initiatives	Low lethality (n,%)	Medium lethality (n,%)	High lethality (n,%)	Total (n,%)
No	4 (23.5%)	4 (36.4%)	2 (28.6%)	10 (28.6%)
Yes	13 (76.5%)	7 (63.6%)	5 (71.4%)	25 (71.4%)
Total	17 (100%)	11 (100%)	7 (100%)	35 (100%)

Fisher's p = 0.890

Initiatives	Low mortality (n,%)	Medium mortality (n,%)	High mortality (n,%)	Total (n,%)
No	3 (27.3%)	5 (33.3%)	2 (22.2%)	10 (28.6%)
Yes	8 (72.7%)	10 (66.7%)	7 (77.8%)	25 (71.4%)
Total	11 (100%)	15 (100%)	9 (100%)	35 (100%)

Fisher's p = 0.903
